# A highly divergent archaeo-eukaryotic primase from the *Thermococcus nautilus* plasmid, pTN2

**DOI:** 10.1093/nar/gkt1385

**Published:** 2014-01-20

**Authors:** Sukhvinder Gill, Mart Krupovic, Nicole Desnoues, Pierre Béguin, Guennadi Sezonov, Patrick Forterre

**Affiliations:** ^1^Institut Pasteur Unité Biologie Moléculaire du Gène chez les Extrêmophiles, 25 rue du Docteur Roux, 75015 Paris, France, ^2^CNRS UMR 7138 Systématique, Adaptation, Evolution, Université Paris 6 quai Saint-Bernard, 75252 Paris Cedex 05, France and ^3^Univ Paris-Sud Institut de Génétique et Microbiologie, CNRS UMR 8621, Orsay 91406, France

## Abstract

We report the characterization of a DNA primase/polymerase protein (PolpTN2) encoded by the pTN2 plasmid from *Thermococcus nautilus*. Sequence analysis revealed that this protein corresponds to a fusion between an N-terminal domain homologous to the small catalytic subunit PriS of heterodimeric archaeal and eukaryotic primases (AEP) and a C-terminal domain related to their large regulatory subunit PriL. This unique domain configuration is not found in other virus- and plasmid-encoded primases in which PriS-like domains are typically fused to different types of helicases. PolpTN2 exhibited primase, polymerase and nucleotidyl transferase activities and specifically incorporates dNTPs, to the exclusion of rNTPs*.* PolpTN2 could efficiently prime DNA synthesis by the *T. nautilus* PolB DNA polymerase, suggesting that it is used *in vivo* as a primase for pTN2 plasmid replication. The N-terminal PriS-like domain of PolpTN2 exhibited all activities of the full-length enzyme but was much less efficient in priming cellular DNA polymerases. Surprisingly, the N-terminal domain possesses reverse transcriptase activity. We speculate that this activity could reflect an ancestral function of AEP proteins in the transition from the RNA to the DNA world.

## INTRODUCTION

Genes encoding DNA-dependent DNA polymerases (hereafter referred to as simply DNA polymerases) are present in all cellular genomes (with often several functional analogues and/or paralogues) and in genomes of many double-stranded DNA viruses. Most of these DNA polymerases require a free 3’OH borne by DNA strands or RNA primers to initiate DNA synthesis. The RNA primers are synthesized by specific DNA-dependent RNA polymerases called DNA primases, which are also ubiquitous in the living world. Notably, some DNA primases exhibit DNA-dependent DNA polymerase activity, and could be considered as novel classes of DNA polymerases (primases/polymerases). Whereas DNA polymerase and/or primase genes are systematically present in cellular genomes and frequently found in the genomes of large DNA viruses, they are apparently much less frequent in plasmids. For a long time, the only plasmids known to encode DNA polymerases were double-stranded DNA plasmids of fungal mitochondria (1). In 2003, Georg Lipps identified a DNA polymerase/primase-encoding gene in a small cryptic plasmid, pRN1, from the thermophilic archaeon, *Sulfolobus islandicus* (2). Interestingly, this DNA polymerase/primase can prime DNA synthesis on a single-stranded template without addition of a 3’OH-containing oligonucleotide as primer, indicating that it can synthesize a ‘DNA primer’. The pRN1 DNA polymerase/primase is a large enzyme, corresponding to the fusion of a DNA polymerase/primase domain with a superfamily III helicase domain. Structural analyses of the DNA polymerase/primase domain have shown that it is evolutionarily related to the catalytic subunit (PriS) of the archaeal and eukaryotic primase (3). Further sequence analyses have shown that these proteins belong to a wide superfamily of proteins, which was termed the archaeo-eukaryotic primase (AEP) superfamily (4).

Recently, we identified a novel DNA polymerase-encoding gene in two cryptic plasmids, pTN2 from *Thermococcus nautilus* and pP12-1 from *Pyrococcus* sp.12-1 (5). The DNA polymerase activity of the pTN2 DNA polymerase (hereafter called PolpTN2) was validated experimentally (5). Originally (5), BLAST search using PolpTN2 as a query retrieved a few hits to proteins encoded by archaeal genomes: two very similar *Methanococcales* proteins (MvolDRAFT_1375 and MvolDRAFT_1398) encoded by the A3-VLP provirus of *Methanococcus voltae* A3 (6) and the putative Rep protein of the pXZ1 plasmid from *S. islandicus* (7). Alignment of these sequences allowed us to identify four conserved motifs in the N-terminal moiety of the enzyme, including a DhD motif that is present in many DNA polymerases and exonucleases. More recently, we could identify homologues of PolpTN2 in five new *Thermococcales* plasmids and one *Methanococcales* plasmid from *Methanocaldococcus vulcanius* M7 (8). Some of these plasmids encode homologues with sizes similar to the full length polymerase, whereas others encode half-sized homologues corresponding either to the N-terminal or the C-terminal half of PolpTN2, suggesting that, as in the case of the pRN1 DNA polymerase/primase, the PolpTN2 enzyme is formed by the association of two domains (8).

Here, we report further *in silico* analysis and biochemical characterization of PolpTN2. Sequence similarities and secondary structure prediction indicate that this enzyme is the prototype of a new polymerase/primase family within the AEP superfamily. PolpTN2 is indeed formed by the association of an N-terminal DNA polymerase/primase domain and a C-terminal domain whose sequences bear a distant similarity to the catalytic and regulatory subunits, respectively, of heterodimeric (PriS–PriL) archaeal and eukaryotic primases. As expected from these *in silico* analyses, we observed that PolpTN2 exhibits DNA polymerase, DNA primase and nucleotidyl transferase activities. Notably, PolpTN2 can efficiently prime DNA synthesis by cellular DNA polymerases, suggesting that this protein is used *in vivo* as primase for pTN2 plasmid replication.

To better understand the functions of the two domains of PolpTN2, a shorter form of this enzyme was engineered by removing the PriL-like domain. The truncated enzyme was less susceptible to proteolysis than the native one and retained the DNA polymerase, primase and nucleotidyl transferase activities of the intact protein, confirming that the catalytic activity resides in the N-terminal PriS-like domain. However, the truncated protein was only poorly active in priming the synthesis of double-stranded DNA. Intriguingly, the truncated protein displayed a reverse transcriptase activity, which was not observed with the intact enzyme. Together, these results support the hypothesis that the PriL-like domain enhances the stringency of PolpTN2 polymerase.

## MATERIALS AND METHODS

### Sequence analysis

Homologues of PolpTN2 were searched for in the non-redundant protein database at the National Center for Biotechnology Information using PSI-BLAST with an upper threshold E-value of 0.05 (9). The searches were seeded with either full-length or fragments of the PolpTN2 sequence. The results from each of the PSI-BLAST iteration were carefully examined prior to launching the next round of iterations. Searches for structural homologues were performed using HHpred (10). Secondary structure was predicted using Jpred3 (11) and PsiPred (12). The multiple sequence alignments were constructed using PROMALS3D (13,14) and following manual adjustment visualized using JalView (15).

### Chemicals and enzymes

Radiolabelled nucleotides were purchased from Perkin Elmer. The unincorporated radiolabel was removed using Microspin™ G-50 columns from GE Healthcare. Unlabelled dNTPs and NTPs were purchased from Sigma Aldrich; T4 polynucleotide kinase (PNK) and restriction enzymes were bought from Fermentas. Phusion polymerase was from Finnzymes. AMV reverse transcriptase and *Taq* polymerase were purchased from Promega and Thermo Fisher Scientific.

### Production and purification of PolpTN2 and PolpTN2Δ_311-923_

Wild-type PolpTN2 protein was expressed and purified as described previously (5) with the following changes: the coding sequence was cloned into the pDEST17 plasmid (Invitrogen), induction at 14°C overnight with 1 mM IPTG (Sigma Aldrich) was performed when the cell culture reached an OD_600_ of 1.0 and the cells harvested from 1 L of culture were re-suspended in buffer A (50 mM Tris–HCl pH 8.0, 300 mM NaCl, 5 mM β-mercaptoethanol). After nickel-nitrilotriacetic acid (Ni-NTA) chromatography of the heat-treated extract, sodium dodecyl sulphate polyacrylamide gel electrophoresis (SDS-PAGE) analysis showed that the flow-through contained the bulk of intact protein, whereas the fractions eluted with imidazole contained mainly degraded protein. The flow through was brought to 70% ammonium sulfate saturation by adding ground ammonium sulfate and stirring constantly at 4°C. Once the ammonium sulfate was completely dissolved, the precipitation was allowed to continue for 30 min. The precipitated protein was centrifuged at 43 000g for 30 min in a pre-cooled rotor, and the resulting pellet was dissolved in 10 ml of 50 mM Tris–HCl pH 8.0, 400 mM NaCl and dialyzed overnight against 50 mM Tris-HCl pH 8.0, 150 mM NaCl. The dialysed preparation was treated for 1 h at 4°C with streptomycin sulfate (21 mg/ml final concentration) and centrifuged at 43 000g for 30 min. The resulting pellet containing PolpTN2 co-precipitated with nucleic acids was re-suspended in 10 ml of 1× PBS. After a second round of centrifugation, the pellet was re-suspended in 5 ml of 1× PBS and 1/10th volume of 5 M NaCl (final concentration, 0.9 M NaCl). A third centrifugation step was carried out at 43 000g for 30 min. The resulting supernatant contained the bulk of intact PolpTN2 protein, leaving the nucleic acids in the pellet bound to some PolpTN2. This supernatant was subsequently loaded onto a Superdex 16/60 200 column (Amersham Pharmacia Biotech) equilibrated against buffer A. The homogeneity of the protein sample was checked by SDS–PAGE. The PolpTN2Δ_311__–923_ construct was obtained using specific primers (851 forward and 851 reverse, refer to Supplementary Table S1) to amplify a 933-bp fragment of the Tn-12p gene that was subsequently cloned between the NdeI and XhoI sites of the pET30 vector. Expression of the PolpTN2Δ_311__–923_ protein was carried out as described above with the following changes: induction was carried out at 37°C for 4 h. The streptomycin sulfate treatment was omitted as the expressed protein was not degraded and could be purified in homogeneous and stable form on the Ni-NTA column.

### Production and purification of *T. nautilus* PolB polymerase

To remove the two inteins encoded by the native *T. nautilus polB* gene, which extend from C_407_ to N_766_ and from S_904_ to N_1289_, the three segments of the gene comprising nucleotides 4–1218, 2299–2709 and 3868–4566, which encode the regions flanking the inteins, were amplified with oligonucleotide pairs PolB-int 0004-PolB-int 1244r, PolB-int 1217-PolB-int 1641r and PolB-int 1621-PolB-int 2322r (Supplementary Table S1), respectively. The three amplified segments were then purified and joined by PCR overlap extension. The reconstituted sequence starting at the second codon of the gene and ending at the stop codon was inserted between the Klenow polymerase-filled NcoI site and the BamHI site of the pETM-11 plasmid (EMBL Protein Expression and Purification Facility). The recombinant plasmid was introduced into *Escherichia coli* SoluBL21(DE3) (Genlantis) bearing the pCodonPlusRIPL plasmid (Agilent Technologies), which provides an adequate supply of rare tRNAs.

Production was performed by cultivating the strain at 37°C in 2YT medium containing 30 µg/ml kanamycin and 30 µg/ml chloramphenicol. The culture was induced with 1 mM IPTG at an OD_600_ of 1, followed by further incubation for 3 h at 37°C. Frozen cell pellets from 750 ml of culture were re-suspended in 18 ml buffer B (50 mM NaHPO_4_, pH 7.8, 400 mM NaCl, 10 mM imidazole) containing one tablet per 20 ml of protease inhibitor cocktail (Roche cOmplete Ultra mini). Cells were broken by two passages at 100 MPa in a French pressure cell, and the crude extract was cleared by centrifugation. The supernatant was loaded on a 2-ml Talon Superflow Co^2+^-agarose (Clontech) column equilibrated with buffer B. After extensive washing with buffer B, the protein was eluted with the same buffer containing 300 mM imidazole. Lower molecular mass impurities owing to proteolytic degradation were removed by dialyzing the preparation against 25 mM NaHPO_4_ pH 7.8, leading to a precipitation of the protein. This was followed by extraction of PolB with the same buffer containing 120 mM NaCl. The intact protein was thus largely separated from degradation fragments, whose solubilization required higher salt concentrations.

### Nucleic acid substrates

Oligonucleotides were synthesized and purified by Sigma Aldrich. Oligonucleotide A45 (refer to Supplementary Table S1) were blocked at their 3’ end with a spacer C3 phosphoramidite. Spacer C3 is a three-carbon spacer. When incorporated at the 3’-end of an oligonucleotide, it blocks extension of the oligonucleotide by polymerase or terminal transferase efficiently. Oligonucleotides were radiolabelled as follows: primers (100 nM) were incubated with [γ-^32^P] ATP (10 μCi), T4 polynucleotide kinase (1 U) in 1× T4 polynucleotide kinase buffer for 30 min at 37°C. The T4 polynucleotide kinase was inactivated by incubation at 80°C for 15 min. The total reaction volume was 10 μl and free [γ-^32^P] ATP was removed using Microspin™ G-50 columns

### Non-radioactive primase and DNA polymerase assays

Reactions were carried out in *Taq* polymerase buffer (75 mM Tris-HCl pH 8.8 at 25°C, 20 mM (NH_4_)_2_SO_4_, 0.01% Tween 20) (Thermo) containing 2.5 mM MgCl_2_, 0.4 mM of each of the four dNTPs, 2 ng/µl of M13mp18 single-stranded DNA (New England Biolabs) and enzyme components as described in the text. Mixes containing all components minus enzyme(s) and enzyme(s) diluted in *Taq* buffer + MgCl_2_ were preheated to 70°C separately before mixing and incubating further at 70°C. At indicated time intervals, aliquots were withdrawn and quenched into microtiter wells containing 25 mM Na-EDTA, pH 8. Double-stranded DNA synthesized was assayed by adding 1 vol Sybr® Green I (Invitrogen) diluted 1/2500 and measuring fluorescence at 520 nm, with excitation at 480 nm, in a Tecan Infinite 200 microplate reader. Fluorescence data were converted into double-stranded DNA concentrations using a standard curve constructed using dilutions of a linear double-stranded DNA fragment whose concentration had been determined from its A_260_. Primase activity was determined using M13 DNA without primer; primer-dependent DNA polymerase activity was determined after hybridizing the M13mp18 DNA template with M13 forward primer (refer to Supplementary Table S1). For this, the template (25 µg/ml) and the primer (1 µM) were heated together for 3 min at 75°C in 0.8 X *Taq* buffer + 2 mM MgCl_2_, followed by cooling to 15°C at 0.1°C/s using a PCR machine.

### Radioactive primase assays

Unless stated otherwise, primase assays were carried out in a total volume of 20 μl. PolpTN2 or PolpTN2Δ_311-923_ (1 µM) was incubated in primase buffer (50 mM Tris pH 8.0, 10 mM MgCl_2_ and 1 mM β-mercaptoethanol) with 5 μM of different oligonucleotides blocked at the 3′ end (refer to figure legends) in the presence of 0.6 nM [α-^32^P] dTTP or dATP and 10 μM (d)NTPs. The reactions were incubated for 30 min at 70°C. An equal volume of stop buffer [98% (v/v) formamide, 10 mM EDTA (pH 8), 1 mg/ml bromophenol blue] was added to the reaction. The reaction products were then separated on a 16% denaturing polyacrylamide gel.

### Terminal transferase assays

Twenty nanometre substrates labelled with ^32^P were incubated in 20 mM Tris–HCl pH 7.5 with 1 µM PolpTN2 or PolpTN2Δ_311__–923_, 100 µM non-labelled dNTPs and 10 mM of Mg^2^ at 60°C for 30 min. The reactions were stopped with the addition of 1 volume of stop buffer [98% (v/v) formamide, 10 mM EDTA (pH 8), 1 mg/ml bromophenol blue] and boiled for 3 min. The samples were subsequently resolved on a 16% polyacrylamide denaturing gel, and radioactivity was detected by autoradiography.

### Reverse transcriptase assays

To test for reverse transcriptase activity, a 30-mer RNA template (30 RT—Supplementary Table S1) was annealed to a radiolabelled 20-mer primer (20 RT—Supplementary Table S1). Reactions were carried out in a volume of 20 μl. The buffer used was 50 mM Tris-HCl pH 8.5, 30 mM KCl and 8 mM MgCl_2_. RNasine (40 U) was included in the assay to protect against any RNases. Five nanomolar of primer–template hybrid, 250 μM of dNTPs and 1 μM of protein were added. Thirty-minute reactions were carried out at 65°C for PolpTN2 and PolpTN2Δ_311__–__923_ and at 42°C for AMV reverse transcriptase. The reactions were quenched on ice and with addition of 20 μl of stop buffer containing formamide, as described above.

### cDNA synthesis and RT fidelity assay

The fidelity assays were performed as described previously (16) with some modifications. *lac*Zα RNA for cDNA synthesis was produced *in vitro* by T7 RNA polymerase from New England Biolabs (NEB) and plasmid pBSC SK—linearized with PciI (NEB). The RNA was purified using Nucleospin RNA II-binding spin columns (Macherey–Nagel) and dissolved in RNase-free water. 500 ng of primer lacZ-rev (Supplementary Table S1) was annealed to 2 µg of *lacZ*α RNA by incubation at 60°C for 5 min followed by 10 min cooling at room temperature. cDNA synthesis reactions contained 11 µM PolpTN2Δ_311__–__923_ or 10 U AMV reverse transcriptase (Promega) and 10 mM total dNTPs with the appropriate buffer for each enzyme (10× buffer: 250 mM Tris-HCl pH 8.3, 250 mM KCl, 50 mM MgCl_2_, 2.5 mM spermidine and 50 mM DTT). Reactions were incubated for 1 h at 60°C for PolpTN2Δ_311__–__923_ and at 42°C for AMV RT. RNase A/T1 mix (Thermo Scientific) was added to the RT reactions, and reactions were incubated for 60 min at 37°C followed by 10 min at 80°C. The cDNA was purified using a PCR clean-up kit (Macherey–Nagel) and amplified with primers IP1 and IP2 (see Supplementary Table S1) and Phusion DNA polymerase. PCR was performed for 30 cycles consisting of 98°C for 30 s, 58°C for 30 s and 72°C for 15 s. Primers IP1 and IP2 introduced ends homologous to the pBC SK-vector. A vector fragment devoid of the 296-bp *lacZ* fragment was generated by inverse PCR amplification of the pBC SK-vector using the divergent primers VP1 and VP2. Both insert and vector PCR products were treated with 20 u DpnI to destroy original templates, and purified on spin columns (Macherey Nagel). The InFusion cloning kit (ClonTech) was used as per manufacturer’s instructions to seamlessly fuse the insert and vector. The circular DNA was transformed according to the manufacturer’s protocol (New England Biolabs) into NEB 10-beta competent cells, which were plated on X-Gal-IPTG plates. Mutation frequency was determined by dividing the number of LacZ^-^ (white) clones by the total number of colonies. The synthesis of cDNA, cloning and counting of white colonies were repeated three times for each enzyme. The frequency of background mutations, defined as LacZ^-^ mutations not occurring within the cloned cDNA, was determined by sequencing 32 mutant colonies for AMV reverse transcriptase and 30 mutant colonies from the PolpTN2Δ_311__–__923_ experiments.

### Electrophoretic mobility shift assay

The ^32^P-labelled 30-mer ribonucleotide 30 RT and its DNA homologue 30 DNA (Supplementary Table S1) (7.5 nM each) were incubated for 30 min at 65°C with increasing concentrations of PolpTN2 or PolpTN2Δ_311__–__923_ in 50 mM Tris-HCl buffer, pH 8, containing 20 mM KCl and 10 mM MgCl_2_, followed by non-denaturing electrophoresis on a 10% polyacrylamide gel in TBE buffer and autoradiography.

## RESULTS

### PolpTN2 sequence analysis

**Figure 1.**
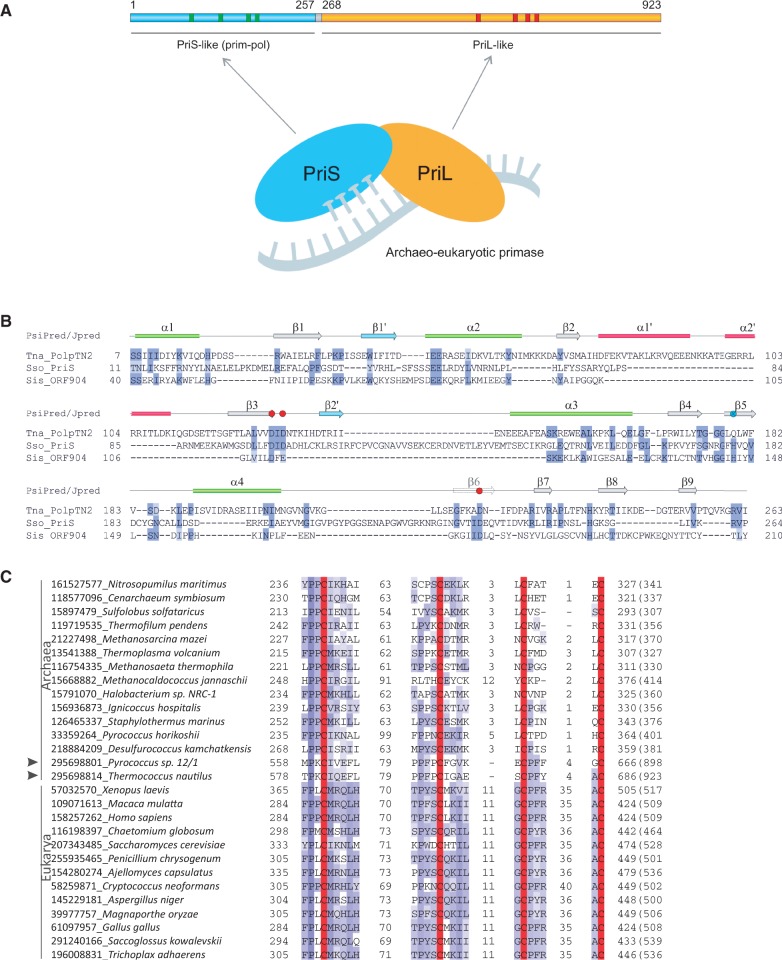
Sequence analysis of the N-terminal PriS-like domain of PolpTN2. (**A**) Domain organization of the PolpTN2 primase–polymerase. The four conserved motifs of the Prim–pol domain and the four cysteine residues predicted to be involved in the Fe–S cluster formation are indicated with green and red bars, respectively [Figure adapted from (17)]. (**B**) Alignment of PolpTN2 with with the sequences of *S. solfataricus* PriS (PDB ID: 1ZT2) and the PriS-like domain of ORF904 from *S. islandicus* plasmid pRN1 (PDB ID: 3M1M). The alignment is coloured according to sequence conservation (BLOSUM62 matrix). The secondary structure of PolpTN2 is indicated above the alignment and was predicted using Jpred and PsiPred. Green ellipses and grey arrows represent respectively α-helixes and β-strands characteristic to the canonical AEP fold (4), while red ellipses and blue arrows represent α-helixes and β-strands that are specific to PolpTN2. Negatively and positively charged residues that constitute the active site of PriS-like proteins are depicted with red and blue circles, respectively (note that the catalytic His residue present in the β5 strand in canonical AEP superfamily proteins is not conserved in PolpTN2 [crossed blue circle]). (**C**) Multiple sequence alignment of the predicted Fe–S cluster binding motif of PolpTN2 with those found in the large subunits of the archaeal and eukaryotic primases. The alignment is coloured according to sequence conservation (BLOSUM62 matrix). The four conserved Cys residues responsible for coordination of the Fe–S cluster are highlighted in red. The limits of the depicted motifs are indicated by the residue positions on each side of the alignment, with the total length of the protein given in parenthesis. Numbers between the motifs indicate the spacing between the corresponding motifs. The sequences are indicated with their GenBank identifiers followed by the corresponding organism name.

To understand the relationship of PolpTN2 to other proteins involved in nucleic acid metabolism, its sequence was subjected to *in silico* analysis. BLASTP analysis of the full-length protein sequence did not reveal other homologues that would contain the same domain organization, except for proteins from archaeal plasmids related to pTN2 (5,8). Nevertheless, partial hits matching the N-terminal and central regions of PolpTN2 were obtained. These were further explored by using distinct fragments of the PolpTN2 sequence as queries in subsequent PSI-BLAST searches. As discussed below, the results suggested that PolpTN2 consists of two domains corresponding to the PriS and PriL domains, respectively, of heterodimeric AEP primases (Figure 1A).

#### N-terminal PriS-like domain

Following three PSI-BLAST iterations, a number of significant hits (*n* > 40, E < 0.05) were retrieved for the N-terminal region (aa 1–267) of PolpTN2. The majority of sequences were of bacterial or bacteriophage origin, annotated as hypothetical or replication-associated proteins. In agreement with the previous analysis (5), only few hits to archaeal proteins were obtained. Interestingly, the fourth PSI-BLAST iteration resulted in a hit to the catalytic small subunit of the primase (PriS) of *Methanopyrus kandleri* AV19 (NP_613871; E = 3e-04; 19% identity). This result was further substantiated by a hidden Markov model-based HHpred analysis (10), in which PriS of *Sulfolobus solfataricus* (PDB ID: 1ZT2) was found to be the closest structural homologue of the N-terminal region of PolpTN2 (94.4% probability). Notably, even after ten PSI-BLAST iterations, the sequence of the primase/polymerase ORF904 from *S. islandicus* plasmid pRN1 could not be retrieved. This indicates that the two archaeal plasmid-encoded replication proteins (PolpTN2 and ORF904) are not recognizably similar, even though both are related to archaeal PriS proteins (3). PriS-like proteins of the AEP superfamily (4) display a characteristic topology with two nearly orthogonal β-sheets hosting the active site and surrounded by a set of α-helixes (3,17–19). To gain further insights into the relationship between PolpTN2 and PriS-like proteins, we compared the profile of predicted secondary structure elements of PolpTN2 to those calculated from the X-ray structures of *Sso* PriS (17) and pRN1 ORF904 (20). As is evident from Figure 1B, all secondary structure elements constituting the core structural fold of PriS-like proteins are conserved in PolpTN2, indicating that the N-terminal domain of PolpTN2 is likely to adopt the same overall topology. Importantly, the three acidic active site residues responsible for coordination of catalytic metal ions in AEPs are also present in PolpTN2 (Asp128, Asp130 and Asp221) at equivalent positions. Interestingly, however, the active site histidine (His145 in ORF904), which is characteristic of AEPs and has been shown to be essential for the enzymatic activities of pRN1 ORF904 (3,4), is not conserved in PolpTN2 (Figure 1B). The other notable differences between PolpTN2 and PriS-like proteins include several insertions in PolpTN2—two α-helices and two β-strands—with respect to the idealized AEP fold (Figure 1). Of these, the β1’ strand is present in the *Sso* PriS, but not in the pRN1 ORF904. Furthermore, PolpTN2 lacks a Zn-binding motif, which is present in PriS and ORF904 proteins, albeit at radically different locations. Notably, the Zn-binding motif has been shown to be dispensable for the *Sso* PriS activity (17). Consequently, the N-terminal domain of PolpTN2 represents a highly divergent member of the AEP superfamily.

#### C-terminal PriL-like domain

Further PSI-BLAST analysis revealed a PolpTN2 region (aa 283–700) displaying sequence similarity to the large non-catalytic subunit of archaeal/eukaryotic primases (PriL). Surprisingly, the initial hit was obtained to PriL of a fungus, *Cryptococcus neoformans* (XP_567348; 22% identity over 409 aa region). Following inclusion of the *C*. *neoformans* PriL sequence for PSI-BLAST profile calculation, a number of hits were obtained to eukaryotic PriLs after one PSI-BLAST iteration. Hits to archaeal homologues were obtained only after three iterations (hit to PriL of *Methanosaeta thermophila* PT [YP_843453], 16% identity over 230 aa region). Consistently, secondary structure predictions indicate that, as in the case of cellular PriLs, the C-terminal domain of PolpTN2 is rich in α-helixes (data not shown). To get a better understanding of the sequence conservation patterns in PolpTN2 and PriLs, a multiple alignment including both archaeal and eukaryotic sequences was generated (Supplementary Figure S1). It became apparent that positions conserved between the C-terminal domain of PolpTN2 and PriLs extend throughout the alignment and encompass both the N- and C-terminal domains of PriLs (NTD and CTD, respectively). Importantly, the four cysteine residues coordinating a 4Fe-4S cluster in the CTD of eukaryotic and archaeal PriLs (21–23) are also conserved in PolpTN2 (Figure 1C). It therefore appears that the N-terminal and C-terminal domains of PolpTN2 represent a fusion of PriS- and PriL-like domains, respectively.

Proteins of the AEP superfamily typically operate in association with other protein partners or functional domains fused to their C-termini. In archaea and eukaryotes, the DNA primase is a heterodimer consisting of the catalytic subunit, PriS, and the large non-catalytic subunit, PriL. In contrast, virus- and plasmid-encoded PriS-like domains are typically fused to different types of helicases (4). This is, for example, the case of pRN1 ORF904 in which the N-terminal PriS-like domain is fused to the superfamily III (SFIII) helicase domain (2). Analysis of the PolpTN2 sequence following the N-terminal PriS-like domain revealed no recognizable NTP-binding motifs characteristic of helicases (24). This is consistent with the observation that pTN2 plasmid encodes a dedicated SFI helicase (Béguin *et al.*, manuscript in preparation) immediately downstream of the PolpTN2 gene (5).

### Purification of PolpTN2, PolpTN2Δ_311__–__923_ and PolB

**Figure 2.**
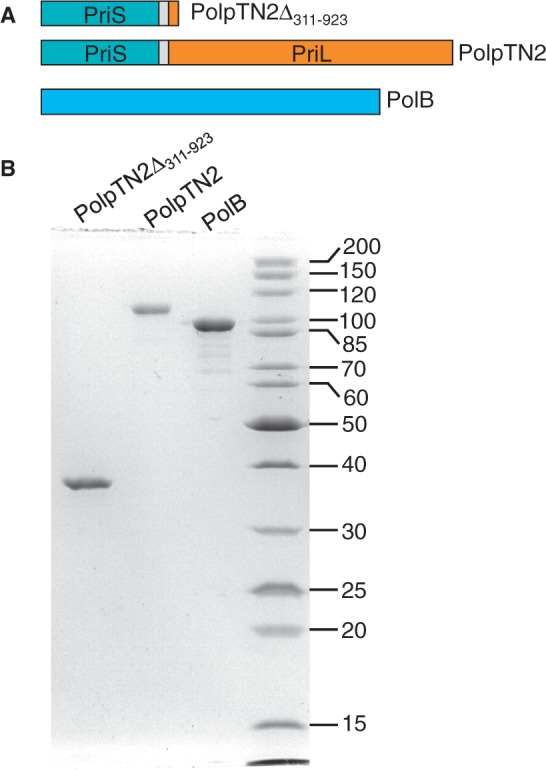
PolpTN2Δ_311–923_, PolpTN2 and Thermococcus nautilus, PolB proteins. (**A**) Schematic representation of the polypeptides; see also Figure 1. (**B**) SDS-polyacrylamide gel analysis of purified PolpTN2Δ_311–923_ (0.5 µg), intact PolpTN2 (0.25 µg) and PolB (0.6 µg).

Initial analysis indicated that the recombinant PolpTN2 protein was produced as a mixture of intact polypeptide and various degradation products, with a major species of about 35 kDa, which MALDI-TOF analysis confirmed that it was derived from PolpTN2 (data not shown). Because sequence and mutational analysis (data not shown) suggested that the catalytic domain lay in the N-terminal domain of the polypeptide, we generated the His-tagged truncated versions PolpTN2Δ_271__–__923_, PolpTN2Δ_311__–__923_ and PolpTN2Δ_360__–__923_. Of these, PolpTN2Δ_311__–__923_ (Figure 2A) could be purified by Ni-NTA affinity chromatography as a non-degraded and catalytically active species and was chosen for further investigation. The full-length PolpTN2 (Figure 2A) was purified taking advantage of the fact that degradation products were preferentially adsorbed on a Ni-NTA column, leaving most of the intact protein in the flow-through. Apparently, in the intact protein, the His tag is less accessible and binds poorly to the column. The purified intact PolpTN2 had a light brown colour, which was consistent with the presence of a Fe–S cluster. The truncated PolpTN2Δ_311__–__923_ lacks the four conserved cysteine residues and, accordingly, the purified protein lacked the light brown colour (data not shown). Recombinant DNA polymerase PolB (Figure 2A) was produced from the reconstituted intron-free sequence of the chromosomal *polB* gene of *T. nautilus* and purified as described in Materials and Methods. The SDS-PAGE analysis of the three purified proteins is presented in Figure 2B.

### Primase and primer-dependent DNA polymerase activity

**Figure 3.**
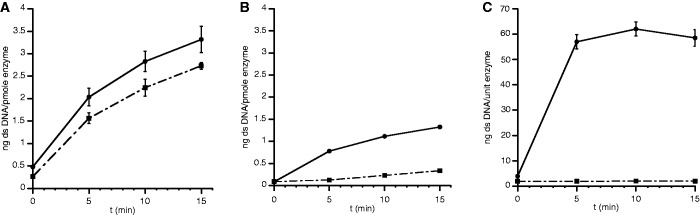
Primase vs. primer-dependent DNA polymerase activity of intact PolpTN2, PolPTNΔ_311–923_ and *Taq* polymerase. (**A**) Intact PolpTN2 (40 ng/µl), (**B**) PolpTN2Δ_311–923_ (80 ng/µl) or (**C**) *Taq* DNA polymerase (0,05 u/µl) were incubated at 70°C in *Taq* buffer + 0,4 mM dNTP and 2 ng/µl M13mp18 DNA, which was or was not hybridized to the complementary oligonucleotide primer M13 forward (Supplementary Table S1). At t = 0, 5, 10 and 15 min, aliquots were withdrawn and quenched into 25 mM EDTA. The amount of ds DNA synthesized was then determined using Sybr® Green I fluorescence as described in Materials and Methods. Circles, continuous line: synthesis with hybridized primer; squares, dashed line: synthesis without primer. Points are the average of two determinations. The standard deviation is indicated by error bars.

As described previously, the PolpTN2 protein possesses DNA-dependent polymerase activity (5). To check whether PolpTN2 protein also exhibits primase activity, as expected from sequence analysis, we used as template single-stranded (ss) M13mp18 DNA with or without hybridized primer. The formation of double-stranded (ds) DNA synthesis was measured following the increase of dsDNA-dependent Sybr® Green I fluorescence. As shown in Figure 3A, the full length PolpTN2 protein was able to synthesize DNA using ss M13 DNA as template without primer in a time-dependent manner, indicating that this protein belongs to the DNA primase/polymerase functional class of enzyme. The presence of a hybridized primer stimulated DNA synthesis only by 15–20%.

The primer-dependent polymerase activity of the PolpTN2Δ_311__–__923_ truncated version, expressed on a per pmole basis, was reduced 2–3 fold as compared with the intact enzyme (Figure 3B). However, the reduction of the primer-independent synthesis activity was much more drastic, so that the ratio between primer-dependent versus primer independent activity was at least 5-fold.

As expected, the activity of *Taq* DNA polymerase was strictly primer dependent in our assay (Figure 3C).

### Priming by intact or truncated PolpTN2 of cellular DNA polymerases

**Figure 4.**
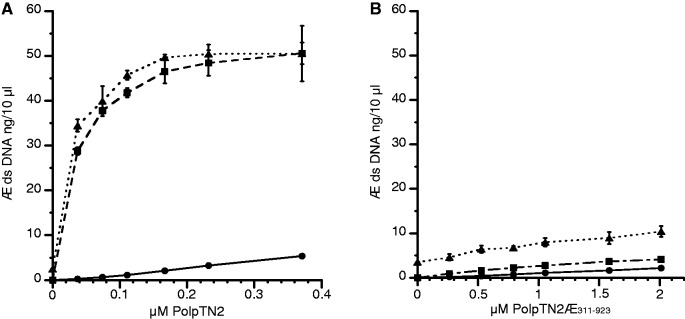
Efficiency of intact PolpTN2 and PolpTN2Δ_311–923_ in priming DNA synthesis by *T. nautilus* PolB and *Taq* DNA polymerase. *T. nautilus* PolB (8 ng/µl) or *Taq* polymerase (0,05 u/µl) were incubated at 70°C in *Taq* buffer + 0,4 mM dNTP and 2 ng/µl M13mp18 DNA (without annealed primer) with increasing concentrations of either intact PolpTN2 (**A**) or PolpTN2 Δ_311–923_ (**B**). DNA synthesis by intact PolpTN2 or PolpTN2 Δ_311–923_ alone was included as a control. At t = 0 and 6 min, aliquots were withdrawn, quenched in 25 mM EDTA and assayed for ds DNA using Sybr® Green I fluorescence. Circles, continuous line: PolpTN2 or PolpTN2 Δ_311–923_ alone; squares, dashed line: PolpTN2 or PolpTN2 Δ_311–923_ plus *Taq* DNA polymerase; triangles, dotted line: PolpTN2 or PolpTN2 Δ_311–923_ plus *T. nautilus* PolB DNA polymerase. Points are the average of three determinations. The standard deviation is indicated by error bars.

To test whether PolpTN2 or its truncated version PolpTN2Δ_311__–__923_ could efficiently prime DNA synthesis by cellular DNA polymerases, we added increasing concentrations of either protein to DNA synthesis assays performed with ssM13mp18 DNA template and *T. nautilus* DNA polymerase PolB or commercial Taq DNA polymerase. Figure 4A shows that low concentrations of intact PolpTN2 strongly stimulated ds DNA synthesis by PolB and *Taq* polymerases. In contrast, we observed only poor priming when PolpTN2 was replaced by PolpTN2Δ_311__–__923_ (Figure 4B). This was true even if the concentration of PolpTN2Δ_311__–__923_ was increased such as to yield more endogenous ds DNA synthesis than was required for nearly optimal stimulation of Taq and PolB polymerases by intact PolpTN (compare endogenous activity, Taq and PolB activities for 2 µM PolpTN2Δ_311__–__923_ with the same activities determined for 0.37 µM intact PolpTN). Some double-stranded DNA synthesis was detected with PolB alone [see first point (triangle) on the abscissa in panels A and B]. This suggests that PolB may display a modest, but significant, self-priming activity. Alternatively, the product detected may arise from primer–template-independent DNA synthesis, as described for *Thermococcus litoralis* (25) and *Thermus thermophilus* (26) DNA polymerases.

### Incorporation of NTPs and dNTPs

**Figure 5.**
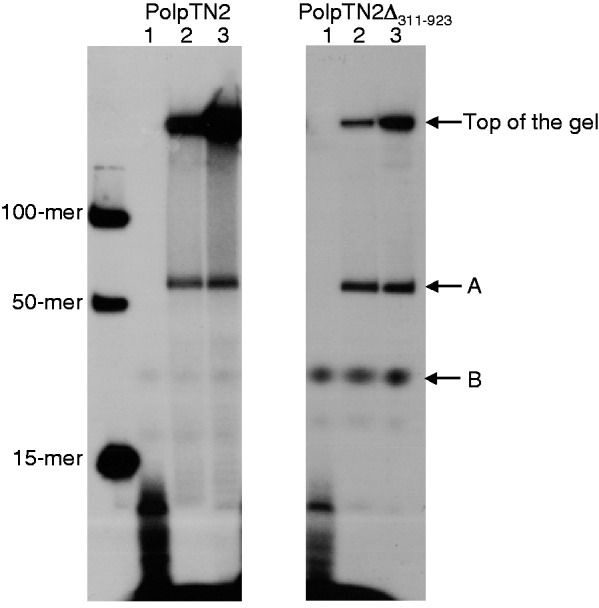
Strictly dNTP-dependent primase activity of PolpTN2 and PolpTN2Δ_311–923_. Primase reactions were performed in a total volume of 60 μl containing primase buffer, 5 μM of oligonucleotide (A45 [Spc C3]), 10 µM dNTPs, 10 µM NTPs, 0.6 nM [α-^32^P] dTTP and 1 µM of protein (PolpTN2 or PolpTN2Δ_311–923_). The reactions were incubated for 30 min at 70°C and subdivided into three 20-µl aliquots. The first aliquot was treated with 2 U of DNase, the second one with 0.3 M KOH and the third one received an equal volume of stop buffer [98% (v/v) formamide, 10 mM EDTA (pH 8), 1 mg/ml bromophenol blue]. The reaction products were then separated on a 16% denaturing polyacrylamide gel. Lane 1: aliquot treated with DNase; lane 2: aliquot treated with 0.3 M KOH; lane 3: aliquot left untreated. Markers are included on the left hand side of the gel (15-mer, 50-mer and 100-mer).

As reported previously (5), PolpTN2 required dNTPs and failed to elongate a labelled primer in the presence of rNTPs. To determine whether NTPs can nevertheless be incorporated during primase activity, we analysed the products synthesized by PolPTN2 and PolpTN2Δ_311__–__923_ in the presence of a mixture of rNTPs and dNTPs. One μM of either protein was incubated with a 45-mer poly-dA oligodeoxynucleotide with a Spacer C3 modification on the 3’ end precluding elongation of the template by terminal transferase activity (refer to Materials and Methods for details), in the presence of 10 μM of rNTPs and dNTPs. The first and second aliquots were treated with 2 U of DNase and 0.3 M KOH, respectively, while the third one received an equal volume of stop buffer, as described in Materials and Methods. Three bands were observed upon denaturing gel electrophoresis of the resulting material (Figure 5). The top bands seen in lanes 2 and 3 (above 100-mer) for both enzymes correspond to products stuck in the loading wells. Two other species, migrating slightly above the 50-mer marker (i) and between the 15-mer and the 50-mer (ii), respectively, were present in the untreated sample generated by PolpTN2Δ_311__–__923_. Species B was nearly absent in the PolpTN2 samples. The nature of these bands is unclear and will need further characterization. They resemble the dAMP–glycerol and dAMP–Tris adducts reported by Chemnitz-Galal *et al.* (27), which are formed by the *T. kodakaraensis* p41 catalytic subunit alone and the *T. kodakaraensis* p41-p46 complex in the absence of a DNA template. As can be observed in Figure 5 lane 1, the DNase treatment shows a clear effect. For both PolpTN2 and PolpTN2Δ_311__–__923_ proteins, it degraded species A and the material at the top of the gel into small fragments, hence confirming that dNTPs are incorporated into these species. By contrast, no shorter fragments were generated in the aliquots treated with KOH (lane 2), indicating that rNTPs were not significantly incorporated into the products [the lesser intensity of the band at the top of the gel in comparison to the untreated control (lane 3) is likely due to a loading artifact].

### Terminal transferase activity

**Figure 6.**
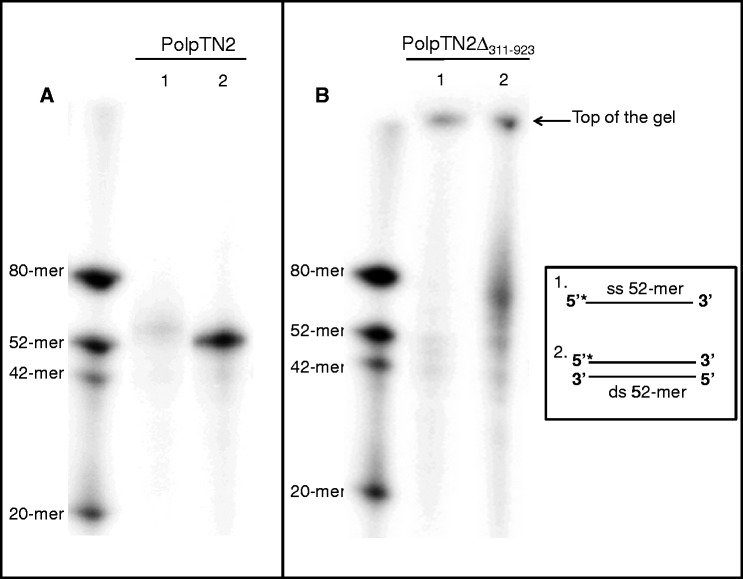
Terminal transferase activity of PolpTN2 (**A**) and PolpTN2Δ_311–923_ (**B**). 5′ ^32^P-labelled substrates were incubated with PolpTN2 or PolpTN2Δ_311–923_ and non-labelled dNTPs in the presence of 10 mM of Mg^2+^ as described in Materials and Methods, followed by denaturing gel electrophoresis and autoradiography. A radiolabelled DNA ladder is included to the left and in the middle of the gel. The ladder also serves as a negative control as it consists of the templates used in the experiment. Templates consisted of **1**: a single-stranded 52-mer (ss TT forward, see Supplementary Table S1) and **2**: a double-stranded 52-mer (ss TT forward annealed to its complementary oligonucleotide ss TT reverse). Asterisks indicate the position of the ^32^P label.

Like the heterodimeric DNA polymerase/primase of *S. solfataricus* (28,29) and the monomeric primase–polymerase domain encoded by the plasmid pIT3 from *S. solfataricus* strain IT3 (30), PolpTN2 displayed terminal transferase activity. To characterize in more detail this activity, we used two different types of primer-templates, i.e single-stranded DNA and blunt double-stranded DNA, in the presence of Mg^2+^(Figure 6A and B) (see Materials and Methods for details). The wild-type PolpTN2 was able to add a sole deoxynucleotide to the single stranded template. No activity was detected with the blunt double stranded DNA (Figure 6A). Figure 6B shows that PolpTN2Δ_311__–__923_ had terminal transferase activity with both templates and synthesized long stretches of DNA in these conditions.

### Reverse transcriptase activity

**Figure 7.**
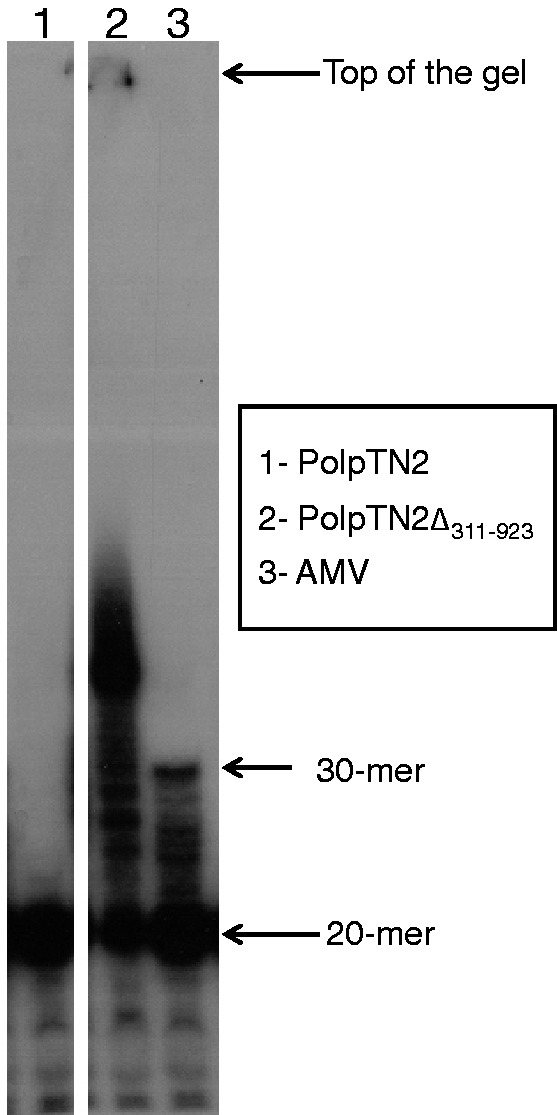
Reverse transcriptase activity of PolpTN2 and PolpTN2Δ_311–923_. A 30-mer RNA template (30 RT, see Supplementary Table S1) was annealed to a radiolabelled 20-mer primer (20 RT). Reactions were carried out in a volume of 20 µl of reverse transcriptase buffer. A control with commercial AMV enzyme was included. Reactions were quenched on ice and with addition of 20 µl of stop buffer containing formamide. 1 = PolpTN2, 2 = PolpTN2Δ_311–923_ and 3 = AMV commercial enzyme. The products longer than the template synthesized by PolpTN2Δ_311–923_ are probably due to the inherent terminal transferase activity of the enzyme.

In addition to the primase, polymerase and terminal transferase activities described here, we tested PolpTN2 and PolpTN2Δ_311__–__923_ for reverse transcriptase activity. Figure 7 shows that, like commercial AMV reverse transcriptase, PolpTN2Δ_311__–__923_ displayed reverse transcriptase activity, while, under the experimental conditions used, the intact PolpTN2 was unable to use RNA as a template.

The combination of primase, polymerase and reverse transcriptase activities in one enzyme would open interesting perspectives for practical applications, provided that the enzyme proceeds with satisfactory fidelity and processivity. Therefore, we used a *lac*Z-based forward mutation assay to measure the error rate of the reverse transcriptase activity of the PolpTN2Δ_311__–923_ enzyme. A fragment of the *lac*Z gene was transcribed *in vitro*, and the resulting *lac*Z mRNA was reverse transcribed into cDNA, which was amplified and cloned again (refer to Materials and Methods). After sequencing a set of mutant clones, the frequency of mutations occurring within the cloned cDNA was calculated by subtracting background mutation frequency from the observed frequency of white colonies. In addition to background mutations not occurring within the cloned segment, sequencing of LacZ^-^ clones obtained from cDNA generated by PolpTN2Δ_311__–__923_ revealed that approximately 25% of mutant clones (8/30) had deletion gaps of approximately 150–250 bases. These deletions were due to poor processivity, with the enzyme failing to synthesize the entire 296 bases of cDNA. The remaining mutants were accounted for by full-length clones harboring point mutations due to misincorporation. The misincorporation rate was calculated by dividing the frequency of such mutants by target size of 296 bases and a mutation detection factor of 0.35 (which represents the fraction of possible nucleotide substitutions generating detectable changes in *lac*Z gene expression (31). Table 1 summarizes the error rates for this study. The error rates for the control AMV reverse trancriptase agree with the rates published by Arezi and Hogrefe (16), albeit slightly lower. The total frequency of mutations generated by PolpTN2Δ_311__–__923_ (1.16* × *10^−1^) was two orders of magnitudes higher than the frequency observed with AMV reverse transcriptase (1.21* × *10^−^^3^). After subtracting the deletion mutations that were generated by PolpTN2Δ_311__–923_, a point mutation rate of 8.2* × *10^−^^4^ was calculated for the enzyme, which was about 70-fold higher than the rate observed for the control AMV reverse transcriptase (1.17* × *10^−^^5^).

**Table 1.** Reverse transcription error rates determined by *lac*Z-based forward mutation assay

Reverse transcriptase	Total mutation frequency	Deletions	Point mutations	Error rate
PolpTN2Δ_311–923_	1.16 × 10^−1^	3.1 × 10^−2^	8.5 × 10^−2^	8.2 × 10^−4 ^nt^−1^
AMV reverse transcriptase	1.21 × 10^−3^	<4 × 10^−5^	1.21 × 10^−3^	1.17 × 10^−5 ^nt^−1^

Clones harbouring a fragment of the *lacZ* gene generated by reverse trancriptase and PCR amplification of the cDNA were tested for their Lac phenotype by plating on X-Gal-IPTG plates. The true mutation frequency was calculated after correcting the frequency of Lac^-^ colonies (white) for the frequency of Lac^-^ carrying no mutation in the cloned fragment. For this, 30 and 32 white clones from the PolpTN2Δ_311–923_ and AMV reverse transcriptase assays respectively, were sequenced. The frequency in the whole population of mutations occurring in the cloned fragment, and among them the frequency of deletions and point mutations, was calculated by multiplying the fraction found in the sequenced sample by the frequency of white clones. 20 627 and 30 600 clones were analysed for the PolpTN2Δ_311–923_ and AMV reverse transcriptase assays, respectively. The background of white clones bearing no mutation in the insert was 2.32 × 10^–2^ for PolpTN2Δ_311–923_ and 4.28 × 10^–3^ for AMV reverse transcriptase. The error rate due to misincorporation was calculated by dividing the frequency of point mutations by the target size (296 nucleotides) and a mutation detection factor of 0.35 [which represents the fraction of possible nucleotide (nt) substitutions generating detectable changes in *lac*Z gene expression].

### Electrophoretic mobility shift assay

**Figure 8.**
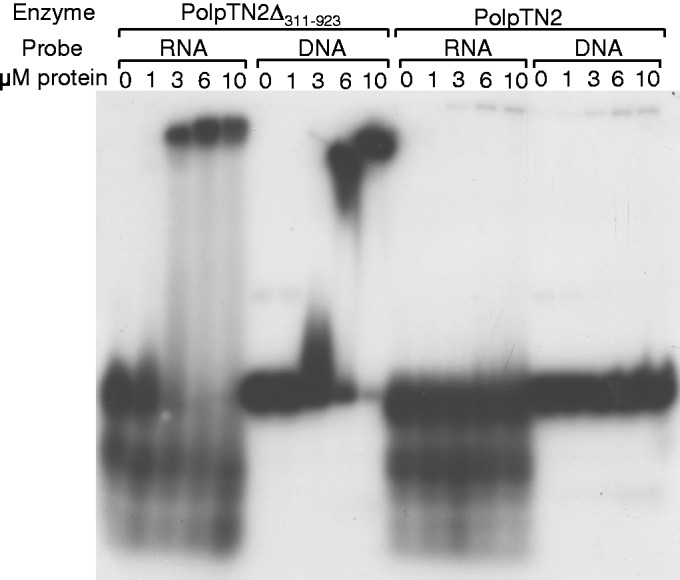
Electrophoretic mobility shift assay. The RNA 30-mer 30 RT and its homologue 30 DNA were incubated with increasing concentrations of PolpTN2Δ_311–923_ and PolpTN2 as described in Materials and Methods. The enzyme, the type of probe and the concentration of each protein are indicated on top of the autoradiogram.

In an attempt to investigate whether the difference between PolpTN2 and PolpTN2Δ_311__–__923_ with respect to reverse transcriptase activity was due to different affinities for single-strand RNA or DNA templates, we performed gel shift assays. The 30-nt RNA template RT 30 and its 30-nt single strand DNA homolog 30 DNA (see Supplementary Table S1) were incubated with increasing concentrations of either protein and subjected to non-denaturing polyacrylamide gel electrophoresis. As shown in Figure 8, a strong retardation was observed for both nucleic acids in the presence of PolpTN2Δ_322__–__923_, whereas, using the same concentrations of protein, very little shifted material could be detected on incubation with the intact enzyme.

## DISCUSSION

Here we have described the sequence features and biochemical characteristics of the replication protein PolpTN2, encoded by the pTN2 plasmid of the hyperthermophilic euryarchaeon *Thermococcus nautilus*. Sequence analysis revealed that the protein is a highly diverged member of the AEP superfamily; all secondary structure elements common to the members of the AEP (4) as well as the catalytic triad (DxD…D) are conserved in the N-terminal, catalytic domain of PolpTN2 (Figure 1). AEP unifies a wide range of functionally versatile proteins involved in nucleic acid synthesis and sharing the characteristic prim-pol topology (4,17,32). It includes the catalytic subunit of archaeal/eukaryotic primases (PriS), PolDom domain of bacterial LigD-like proteins of the NHEJ DNA double-strand break repair pathway (33) as well as replication proteins from bacterial and archaeal viruses and plasmids. A recurrent activity common to all of the experimentally characterized proteins in this group is the ability to synthesize a primer on a DNA template. Indeed, we found that PolpTN2 can also initiate DNA synthesis d*e novo*. However, unlike chromosomal AEP primases, which can polymerize both rNTPs and dNTPs (30,34–36), or ORF904 from *S. solfataricus* plasmid pRN1, which incorporates one rNTP at the first position followed exclusively by dNTPs, the primase activity of PolpTN2 is specific for dNTPs. In this respect, PolpTN2 resembles protein RepB’ from the broad-host range bacterial plasmid RSF1010 (37) and the primase–polymerase of *Brevibacterium* siphovirus BFK20 (38).

PSI-BLAST analysis showed that PolpTN2 is not closely related to any of the previously characterized AEP members, including the ORF904 primase-polymerase encoded by the archaeal plasmid pRN1 (2). Thus, on the basis of sequence divergence, we propose that PolpTN2 should be considered as a prototype member of a new family of primases–polymerases within the AEP. It is pertinent to mention that PolpTN2 does not possess a Zn-binding motif, which is present in archaeal/eukaryotic PriS proteins as well as in ORF904 of pRN1 (3,17–19). Interestingly, in the latter two protein families, the Zn-binding motifs are located at different locations and were likely introduced independently (4). Thus, PolpTN2 might resemble a more ancestral enzyme, which subsequently gave rise to PriS- and ORF904-like enzymes. The possibility that the Zn-binding motif has been lost in PolpTN2 cannot however be excluded.

The similarity of PolpTN2 to the archaeal PriS–PriL primases extends to the C-terminal, PriL-like domain. This is in contrast to the virus- and plasmid-encoded primases in which PriS-like domains are typically fused to different types of helicases (4). Such is the case, for example, with pRN1 ORF904 in which the N-terminal PriS-like domain is fused to the superfamily III helicase domain (2).

A similar fusion of the two primase subunits has only been reported in *Nanoarchaeum equitans* and the unusual *Bacillus* provirus phBC6A51 (4,39). While the *N. equitans* protein has not been studied biochemically, the primase activity of phBC6A51 protein could not be confirmed *in vitro* (40). The catalytic properties of intact and truncated PolpTN2 are fully consistent with the identification of the protein as a PriS–PriL-type primase in which the two subunits are fused into a single polypeptide. As expected, the catalytic activity resides in the N-terminal, PriS-like region. However, removal of the C-terminal, PriL-like domain, strongly affects the catalytic properties of the enzyme. This is consistent with observations made with heterodimeric PriS–PriL primases, which indicate that the non-catalytic PriL subunit modulates the catalytic specificity of PriS. For example, with the *Pyrococcus furiosus* primase, the *Pfu*p41 subunit alone could synthesize long DNA strands. Addition of the non-catalytic subunit *Pfu*p46 enhanced the rate of DNA synthesis, but reduced the size of DNA fragments (35). In the case of the heterodimeric primase of *Pyrococcus abyssi*, the *Pab*p41 subunit alone had no RNA synthesis activity, but could synthesize long DNA strands. Again, addition of the non-catalytic subunit *Pab*p46 enhanced the rate of DNA synthesis, but reduced the size of DNA fragments and enabled the incorporation of ribonucleotides (34). The same pattern was observed with the p41/p46 primase of *Thermococcus kodakaraensis*. Even more to the point, p41 alone primed DNA synthesis by Klenow polymerase only poorly, as compared with priming prompted by the p41–p46 complex (36).

The more efficient priming of DNA polymerases observed with intact PolpTN2 and with the p41–p46 complex as compared with PolpTN2Δ_311__–__923_ and p41 alone may hint that the non-catalytic domain/subunit favours the interaction between the primase complex and the polymerases. This would imply a rather broad specificity of interaction, since the primase complex is able to stimulate incorporation of dNTPs by widely divergent DNA polymerases such as *E. coli* Klenow polymerase and *T. aquaticus* polymerase. Alternatively, the products synthesized by the catalytic domain/subunit alone may not be suitable for elongation by DNA polymerases. This may be the case if, owing to template-independent terminal transferase activity, the 3′ end of the primer fails to hybridize to the template. Indeed, single-nucleotide mismatches at the 3′ end of primers strongly impair elongation by *Taq* DNA polymerase (41). The latter explanation would be consistent with the more relaxed catalytic specificity of the truncated protein. In this respect, it is particularly noteworthy that, contrary to intact PolpTN2, PolpTN2Δ_311__–__923_ acquired reverse transcriptase activity, albeit with low processivity and fidelity. Accordingly, besides ensuring catalytic robustness of PolpTN2, the PriL-like domain apparently contributes to determining the template specificity of the enzyme.

The results of the gel shift assays are in contrast with those reported by Liu *et al.* (35), who found that the catalytic p41 subunit of the dimeric *P. furiosus* primase had only weak DNA-binding affinity as compared with the p46 subunit and the p41–p46 complex. Figure 8 shows that PolpTN2Δ_311__–__923_ displayed a higher affinity than the intact form for both DNA and RNA templates. This might explain why the truncated enzyme was able to act as a reverse transcriptase. Structural analyses will be needed to fully understand the changes brought about by the removal of the C-terminal part of the enzyme.

Catalytic potential varies from one AEP member to the other. Some of the proteins catalyse a single reaction, while others display multiple activities. This is, for example, the case of PriS from *S. solfataricus* (28,29) as well as the primase–polymerase from the *S. solfataricus* plasmid pIT3 (30), which can prime DNA synthesis, elongate the nascent DNA strand in a template-dependent manner and act as non-templated nucleotidyl transferases. Peculiarly, the latter activity could not be demonstrated for the primase of euryarchaeon *Thermococcus kodakaraensis* (36), highlighting the functional variability in even relatively closely related members of the AEP. In addition to being a DNA-synthesizing primase, PolpTN2 also possesses nucleotidyl transferase activity. To our knowledge, the reverse transcriptase activity of the isolated catalytic domain has not been previously demonstrated for any of the AEP members, thus further broadening the spectrum of activities associated with the AEP super family. Reverse transciptase activity is typically associated with proteins that share a set of motifs with retroviral RTs (42). Notably, however, it has been shown that bacterial family A and archaeal family B DNA polymerases may also be engineered to display RT activity (43–45). It is often considered that retroviral-like RTs are remains of the ancient RNA world (46–48) that were at the forefront of the transition from RNA-based information storage to the present-day-like DNA-based genomes. The observation that many of the enzymes currently operating on DNA templates, including DNA polymerases and members of the AEP superfamily, can also act as RT suggests that the ancestors of these respective protein families might have originated and diversified already in RNA-based cells, possibly prior to the emergence of DNA itself. It is tempting to speculate that these proteins, together with retrovirus-like RTs, have played an important role in the transition from the RNA to DNA world.

*In vitro* biochemical evidence indicates that PolpTN2 is an efficient primase. This suggests that PolpTN2 is used as a primase for pTN2 replication in combination with a bona fide polymerase. Our *in vitro* data imply that *T. nautilus* DNA polymerase B, which efficiently interacts with PolpTN2, may play this role. However, this possibility remains to be confirmed by *in vivo* genetic experiments because *T. nautilus* also contains the specific archaeal DNA polymerase D. Another open question, which remains to be clarified, is the role of the SFI helicase whose gene is present in all plasmids of the pTN2 family and is located next to the gene encoding PolpTN2 or relatives. This helicase could be involved in origin opening and/or strand displacement. Indeed, *in silico* analyses have predicted the existence of replication origin in pTN2 (5); however, the biochemical properties of PolpTN2 do not offer an explanation as of how this primase could initiate plasmid replication at a specific site. The pTN2 plasmid appears to be a promising model system to study plasmid DNA replication in hyperthermophilic archaea.

## SUPPLEMENTARY DATA

Supplementary Data are available at NAR Online.

## FUNDING

Direction des Applications de la Recherche of Institut Pasteur (DARRI) [DARRI AO2010 to S.G.]; European Molecular Biology Organization [ALTF 347-2010 to M.K.] (in part). Funding for open access charge: Institut Pasteur.

*Conflict of interest statement*. None declared.

## Supplementary Material

Supplementary Data
