# Supplementary material for: A highly divergent archaeo-eukaryotic primase from the Thermococcus nautilus plasmid, pTN2
Source: Nucleic Acids Res. 2014 Jan 20;42(6):3707–19. doi: 10.1093/nar/gkt1385 (PMC3973330; doi:10.1093/nar/gkt1385)
Supplement: Supplementary Data [file supp_42_6_3707__index.html]

A highly divergent archaeo-eukaryotic primase from the Thermococcus nautilus plasmid, pTN2 — A highly divergent archaeo-eukaryotic primase from the Thermococcus nautilus plasmid, pTN2 — Supplementary Data 

# A highly divergent archaeo-eukaryotic primase from the *Thermococcus nautilus* plasmid, pTN2

## Supplementary Data

files

**Files in this Data Supplement:**

- Supplementary Data - doc file
