# Supplementary material for: A highly divergent archaeo-eukaryotic primase from the Thermococcus nautilus plasmid, pTN2
Source: Nucleic Acids Res. 2014 Jan 20;42(6):3707–19. doi: 10.1093/nar/gkt1385 (PMC3973330; doi:10.1093/nar/gkt1385)
Supplement: Supplementary Data [file supp_gkt1385_nar-02608-v-2013-File011.doc]

### Figure S1**:** Multiple sequence alignment of the C-terminal PolpTN2 domain with the sequences of the large subunit of archaeal and eukaryotic primases.

The sequences are indicated with their GenBank identifiers followed by the corresponding organism names. Conserved residues are highlighted according to their chemical properties using the colour code of ref. (15).

### Table S1. Oligonucleotides used in this study.

| **Primer Name** | **Sequence (5’-3’)** |
| --- | --- |
| 851 forward | CCGCCATATGAGCAGTCTTCGTCCATCTTC |
| 851 reverse | TTTCTCGAGCTAGGACGTGACTTCGTATCTCTTTG |
| PolB-int 0004 | attctcgataccgactacatcaccgag |
| PolB-int 1244r | GTTATGATGATTGAGGGGTACAGCGAcctaaagtctaaatacacaatattgtcccacaatcc |
| PolB-int 1217 | ggTCGCTGTACCCCTCAATCATC |
| PolB-int 1641r | tgtcgcaaagaaaccgtccgtGTCCGCGTAGAGAACTTTAAAGCCG |
| PolB-int 1621 | acggacggtttctttgcgaca |
| PolB-int 2322r | aaaggatccttacttcttcaccttcagccacgc |
| M13 forward | CAGGGTTTTCCCAGTCACGAC |
| ss TT forward | AAAGCTTATAAATAACGAAGAGAGAACAAGATATTAGATAAAAGTTAATAAA |
| ss TT reverse | TTTATTAACTTTTATCTAATATCTTGTTCTCTCTTCGTTATTTATAAGCTTT |
| 30 RT | rAGAGGGUUCAAAAAAUGGUUUCACCCCAAA |
| 30 DNA | AGAGGGTTCAAAAAATGGTTTCACCCCAAA |
| 20 RT | TTTGGGGTGAAACCATTTTT |
| LacZ rev | GCGCAACGCAATTAATGTGAG |
| IP 1 | ATACGACTCACTATAGGGCGAATTGGGTACCGG |
| IP 2 | GAAAGCGGGCAGTGAGCGCAACGCAATTAATGTGAGT |
| VP 1 | TATAGTGAGTCGTATTACGCGCGC |
| VP2 | TCACTGCCCGCTTTCCAGTCG |
| A45 | AAAAAAAAAAAAAAAAAAAAAAAAAAAAAAAAAAAAAAAAAAAAA [SpC3] |
